# Supplementary material for: Identification of photoperiod-induced specific miRNAs in the adrenal glands of Sunite sheep (Ovis aries)
Source: Front Vet Sci. 2022 Jul 22;9:888207. doi: 10.3389/fvets.2022.888207 (PMC9354845; doi:10.3389/fvets.2022.888207)
Supplement: Supplementary file 1 [file Data_Sheet_1.ZIP › Supplementary file/miRDeep2 file.zip!/miRDeep2 file/Novel_Structure/SPLP42A2/Novel_147.pdf]

Provisional ID	:	NC_019484.2_43021
Score total	:	1.9
Score for star read(s)	:	-1.3
Score for read counts	:	0
Score for mfe	:	1.6
Score for randfold	:	1.6
Score for cons. seed	:	
Total read count	:	82
Mature read count	:	82
Loop read count	:	0
Star read count	:	0

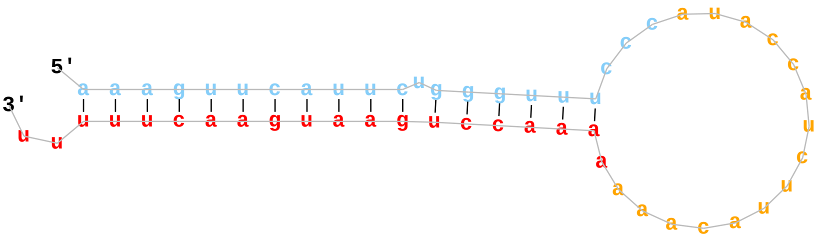

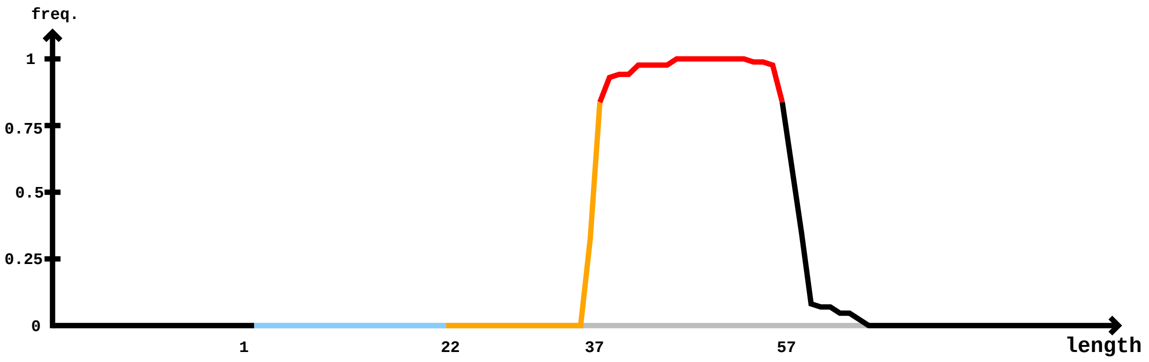

Star Mature

```
5'- gcaguacuggguugcccaaaaaguucauucuggguuucccauaccaucuuacaaaaaaaccugaaugaacuuuuuggccaacucaauauuuauugcgugucuuuguuau   -3'    exp
    ((((((.(((((((.(((((((((((((((.((((((...................))))))))))))))))))))).))))))).....))))))).............    reads    mm    sample
    ......................................................aaaaaccugaaugaaUu......................................    1        1     seq
    ......................................................aaaaaccugaaGgaacuuuu...................................    1        1     seq
    ......................................................aaaaaccAgaaugaacuuuu...................................    10       1     seq
    ......................................................aaaaaccugaaugaacuuuuC..................................    1        1     seq
    ......................................................Caaaaccugaaugaacuuuuu..................................    1        1     seq
    ......................................................aaaaaccugaaGgaacuuuuug.................................    12       1     seq
    ......................................................aaaaaccugaaugaacuuuCug.................................    1        1     seq
    ......................................................aaaaaccugaaugaacuuuuugCc...............................    1        1     seq
    .......................................................aaaaccAgaaugaacuuu....................................    1        1     seq
    .......................................................aaaaccAgaaugaacuuuu...................................    1        1     seq
    .......................................................aaaaccugaaugaacuuuuu..................................    13       0     seq
    .......................................................Uaaaccugaaugaacuuuuu..................................    1        1     seq
    .......................................................aaaaccAgaaugaacuuuuu..................................    7        1     seq
    .......................................................Gaaaccugaaugaacuuuuug.................................    2        1     seq
    .......................................................aaaaccugaaugaacuuuuuA.................................    1        1     seq
    .......................................................aaaGccugaaugaacuuuuug.................................    1        1     seq
    .......................................................aaaaccGgaaugaacuuuuugg................................    1        1     seq
    .......................................................aaaaccugaaugaacCuuuugg................................    7        1     seq
    .......................................................aaaaccugaaugaGcuuuuugg................................    1        1     seq
    .......................................................aaaaccugaaugaacuuuuugU................................    7        1     seq
    .......................................................aaaacUugaaugaacuuuuugg................................    1        1     seq
    ........................................................aaaccugaaugaacuuuuug.................................    1        0     seq
    ........................................................aaaccAgaaugaacuuuuug.................................    1        1     seq
    ........................................................aaaccugaaugaacuuuuugU................................    3        1     seq
    ........................................................aaaccugaGugaacuuuuugg................................    1        1     seq
    ........................................................aaaccugaauAaacuuuuugg................................    1        1     seq
    ........................................................Uaaccugaaugaacuuuuugg................................    1        1     seq
    .........................................................aaccugaaCgaacuuuuuggcca.............................    1        1     seq
    ...........................................................ccugaaugaacuuuuuggccU.............................    1        1     seq
    ...........................................................ccugaaugaacuuuuuggccaaU...........................    1        1     seq
    ...........................................................ccugaaugaacuuuuuggccaaUu..........................    1        1     seq
    ...............................................................aaugaacuuuuuggccaac...........................    1        0     seq
    ...............................................................aaugaacuuuuuggccaacG..........................    1        1     seq
```
